# Apelin-13 modulates the endometrial transcriptome of the domestic pig during implantation

**DOI:** 10.1186/s12864-024-10417-9

**Published:** 2024-05-21

**Authors:** Kamil Dobrzyn, Marta Kiezun, Grzegorz Kopij, Barbara Zarzecka, Marlena Gudelska, Katarzyna Kisielewska, Ewa Zaobidna, Karol G. Makowczenko, Cecilia Dall’Aglio, Tadeusz Kamiński, Nina Smolińska

**Affiliations:** 1 Faculty of Biology and Biotechnology, University of Warmia and Mazury in Olsztyn, Oczapowskiego 1A, Olsztyn, 10-719 Poland; 2 Faculty of Medical Sciences, University of Warmia and Mazury in Olsztyn, Aleja Warszawska 30, Olsztyn, 10-082 Poland; 3 Institute of Animal Reproduction and Food Research of Polish Academy of Sciences, Department of Reproductive Immunology and Pathology, Tuwima 10, Olsztyn, 10-748 Poland; 4 Department of Veterinary Medicine, University of Perugia, Via San Costanzo 4, Perugia, 06126 Italy

**Keywords:** Adipokine, Alternative splicing, Angiogenesis, Apelin, Apoptosis, Differentially expressed genes, Endometrium, RNA-Seq, Transcriptome

## Abstract

**Background:**

The peri-implantation period is a critical time during pregnancy that mostly defines the overall litter size. Most authors agree that the highest percentage of embryo mortality occurs during this time. Despite the brevity of the peri-implantation period, it is the most dynamic part of pregnancy in which the sequential and uninterrupted course of several processes is essential to the animal’s reproductive success. Also then, the maternal uterine tissues undergo an intensive remodelling process, and their energy demand dramatically increases. It is believed that apelin, a member of the adipokine family, is involved in the control of female reproductive functions in response to the current metabolic state. The verified herein hypothesis assumed the modulatory effect of apelin on the endometrial tissue transcriptome on days 15 to 16 of gestation (beginning of implantation).

**Results:**

The analysis of data obtained during RNA-seq (Illumina HiSeq2500) of endometrial slices treated and untreated with apelin (*n* = 4 per group) revealed changes in the expression of 68 genes (39 up-regulated and 29 down-regulated in the presence of apelin), assigned to 240 gene ontology terms. We also revealed changes in the frequency of alternative splicing events (397 cases), as well as single nucleotide variants (1,818 cases) in the presence of the adipokine. The identified genes were associated, among others, with the composition of the extracellular matrix, apoptosis, and angiogenesis.

**Conclusions:**

The obtained results indicate a potential role of apelin in the regulation of uterine tissue remodelling during the peri-implantation period.

**Supplementary Information:**

The online version contains supplementary material available at 10.1186/s12864-024-10417-9.

## Background

The early gestation period is considered the most critical time during pregnancy in mammals. Despite the relatively short time of duration, early gestation, especially the peri-implantation period, is the most dynamic part of the pregnancy, involving several processes, which proper sequence and uninterrupted course are pivotal for the maintenance of pregnancy and, in consequence, the reproductive success of animal. The proper course of pregnancy requires a multitude of correctly synchronised processes that take place in parallel in the embryo and the maternal tissue. The process of attachment of the embryo to the uterine luminal epithelium (LE) can only take place in a short period known as the ‘window of implantation’. The delays in embryo attachment to the LE that lead to skipping this period cause the process failure [1–3]. The peri-implantation period is characterised by the increased endocrine activity of the uterus, involving a significant number of genes and their products. As a result, several factors are secreted, such as adhesive molecules, proteolytic enzymes, or cytokines responsible for the proper course of the implantation process [3]. Despite many factors directly involved in the mechanisms of the maternal recognition of pregnancy and implantation, many other molecules, which are indirectly connected with these processes, are also crucial for pregnancy success. The connection between the reproductive success of animals and their metabolic status has been studied for many years. A growing body of evidence indicates an important role of the adipocytokines, derived mainly from the white adipose tissue (WAT) and involved mostly in the regulation of energy metabolism. The adipokines may also take part in the regulation of reproductive functions in response to the maternal metabolic status.

Apelin, a member of the adipokines group, was primarily isolated from the bovine stomach in 1998 and identified as a ligand of the orphan G protein-coupled receptor, APJ (apelin receptor, also called APLNR) [4]. The hormone is coded by the *APLN* gene located on the human Xq25-26 chromosome and porcine chromosome X. The product of both, the human and porcine gene is a 77-amino acid preproprotein which, through proteolytic cleavage, derives several bioactive peptides such as apelin-36, apelin-17, apelin-13, or pyroglutamate-apelin-13 [4, 5]. Apelin receptor was discovered in 1993 as a G-coupled receptor sharing 40 to 50% homology to angiotensin II receptor type 1. Despite the similarity in the sequence, APJ was found to be unable to bind angiotensin II [6]. The adipokine receptor, depending on which form of apelin binds, activates many signalling pathways, mainly through the activation of ER1/2, PI3, and AMP kinases [7–9]. Apelin was found to exert the pleiotropic effects in the organism. It was reported that the hormone is involved, *inter alia*, in the regulation of cardiovascular system functioning, pressure and blood flow regulation, water, and food intake as well as angiogenesis [10]. Apelin has also been found to be involved in the regulation of important metabolic processes such as insulin secretion [11]. Its stimulatory effect on glucose uptake and glucose transport as well as fatty acid oxidation and its inhibitory effect on lipolysis have also been reported [12–15]. There is a general scarcity of data concerning the hormone actions in the mammalian reproductive system, not only during the time of gestation. The presence of apelin and its receptor genes and proteins in the reproductive structures has been indicated earlier in various tissues of many species, such as human, porcine, bovine and ovine ovaries, ovine oviducts, rat and ovine uteri, as well as in human and canine placentas [16–19]. Our unpublished data indicates the expression of both, apelin and APJ, gene and protein in the porcine endometrium and myometrium, as well as in conceptuses and trophoblasts. However, there is a lack of data concerning in vitro or in vivo effects of apelin on the reproductive structures in mammals. One of a few studies conducted by Rak et al. [18] indicated the modulatory effect of apelin on porcine ovarian steroidogenesis. The hormone increased basal steroid secretion but decreased insulin-like growth factor 1- and follicle-stimulating hormone (FSH)-induced steroid release, as well as the expression of enzymes involved in the steroidogenesis process. In rats, apelin suppressed serum testosterone (T), luteinizing hormone (LH), and FSH concentrations. The results of the few studies presented above in connection to the proven key role of apelin in the regulation of metabolism inspired us to investigate the influence of the hormone on the whole transcriptome of the porcine endometrium during such an energy-demanding period as the time of implantation. We assume that apelin may belong to a group of factors affecting uterine functions during the peri-implantation period through its impact on endometrial gene expression. Therefore, this study aimed to investigate the influence of apelin on the global gene expression and phenomena accompanying the transcription process, such as the alternative mRNA splicing and allele-specific expression variants in the porcine endometrium during implantation. Presentation of the results concerning only the influence of the adipokine on the expression of protein coding genes, would not fully reveal the overall apelin effect on the transcriptomic profile of endometrial tissue. Therefore, we enriched the presented studies with long noncoding RNA, alternative splicing occurrence, and allele specific variants analyses, which let us reveal the comprehensive action of adipokine in the endometrium. Since the short forms of apelin have been indicated to have much higher biological potency than the longer ones [20], in the present study we have investigated the influence of apelin-13 on the porcine endometrial transcriptome. The presented studies were conducted on the porcine endometrial tissue explants acquired during the peri-implantation period, on days 15 to 16 of gestation, which in pigs corresponds to the beginning of implantation. The early gestation period, especially the event of implantation is a very dynamic process that requires the mobilization of a large number of genes and their products, as well as their close cooperation and synchronization. During this period, the endometrial tissue undergoes intensive growth and reconstruction, which includes, among others the creation of new blood vessels, to ensure proper conditions for embryo growth and development (for more see: [3]).

## Methods

### *Animals and* in vitro *endometrial tissue cultures*

Four mature crossbred gilts (Large White × Polish Landrace, age of 7–8 months and 130–140 kg of weight) on days 15 to 16 of gestation (*n* = 4) were maintained and fed following current Polish standards with access to fresh water and forage ad libitum. The animals were descended from a private breeding farm (L. Wisniewski breeding farm, Krolikowo, Poland). The daily dose of energy was approximately 2.7 kg/gilt at 32.4 MJ of metabolizable energy intake (12 MJ per kg of feed). The daily dose of nutrients (g per kg of fed) was as follows: 135 g/kg of total digestible protein, 7.3 g/kg lysine, 5.4 g/kg methionine and cystine, 4.8 g/kg threonine, 2.1 g/kg tryptophan, 8.9 g/kg calcium, 5.7 g/kg total phosphorus, 1.7 g/kg sodium, 10% fibre, and the addition of other macro- and microelements.

All animals were monitored daily for oestrus behaviour and the day of the onset of the second oestrus was recognized as day 0 of the oestrous cycle. The natural inseminations were conducted on the first or second day of the oestrous cycle, and the first day after coitus was marked as day 0 of pregnancy. The animals were inseminated by natural mating using the same, crossbreed boar. The stage of pregnancy was confirmed by the presence and morphology of the conceptuses [21], as well as by determining the levels of P_4_ [22].

The in vitro culture was run on four pregnant gilts. After the animals’ sacrifice, two endometrial samples were retrieved from each uterus. One of them was descended to the control group, whereas the second, was to the adipokine-treated group. The endometrial tissue explants procedure was conducted as described by Dobrzyn et al. [22]. In detail, the tissue explants (irregular slices, weight of 100 mg ± 10% and 3 mm of thickness) were obtained from the middle of the uterine horns and mechanically separated from the myometrium using scissors. Before the procedure conceptuses/trophoblasts were separated from the endometrium and the explants were dissected only from the implantation sites. The slices were preincubated (2 h) and incubated for 24 h in 2 mL of phenol-red free medium M199 (Sigma-Aldrich, USA) in a shaking water bath with gas supply (37 °C, 95% O2, 5% CO2). The tissues from the control groups were incubated with the medium alone, whereas the tissues from the adipokine-treated group were incubated in the presence of human recombinant apelin-13 (Merck, USA; cat. #A6469) at the dose of 20 ng/mL. The dose of adipokine was chosen based on the publications of Rak et al. and Różycka et al. [18, 23]. The in vitro incubations were run in four separate experiments (*n* = 4, one animal for each experiment) in duplicates. The activity of lactate dehydrogenase (LDH) after preincubation and incubation was used to determine the viability of the endometrial tissue explants. The enzyme activity measurement was performed using a Liquick Cor-LDH kit (Cormay, Poland). The LDH activity in the culture media collected after preincubation and incubation was compared with its activity in the fully dissolved tissue (positive control, maximum LDH release). The mean activity of LDH in the culture media after preincubation and 24 h of incubation was as follows: 104 ± 29 U/L (0.55% of maximal LDH release) and 116 ± 36 U/L (0.6% of maximal LDH release), respectively.

### Library preparation and RNA-Seq sequencing

The analysis of the endometrial global gene expression was conducted in one RNAseq experiment and one run for both, the control, and the adipokine-treated groups. The isolation and cleaning-up processes for total RNA were performed using RNeasy Kit and RNase-Free DNase Set (Qiagen, Germany), respectively. The quality and quantity of the obtained RNA were determined using the Tecan Infinite M200 reader (Tecan Group Ltd., Switzerland). Subsequently, all samples were subtracted to the analysis of the RNA integrity number (RIN) using Agilent Bioanalyzer 2100 (Agilent Technology, USA). RNA samples with RINs in the range of 8–10 were used for the transcriptome high-throughput sequencing (RNA-Seq) and subsequent quantitative real-time PCR (qPCR) validations.

Double-stranded cDNA libraries from RNA samples were performed with the use of the Illumina Truseq Standard mRNA LT Sample Prep Kit (Illumina, USA). After the fragmentation of RNA samples, poly(dT) oligonucleotides were used in the process of the strand-specific reverse transcription into cDNA. The obtained cDNA was processed to 3’ tail adenylation and adapter ligation. The transcriptome high-throughput sequencing was performed on the HiSeq2500 platform (Illumina, USA) to generate 2 × 100 bp pair-end reads with an assumed minimal sequencing depth of 100 million reads per sample.

### Transcriptome expression profiling

The *in silico* analyses were mainly performed following the procedure described by Orzechowska et al. [24]. The quality of generated raw reads was determined using FASTQC software (v. 0.11.8; [25]). For the removal of the adapters and low-quality reads Trimmomatic software (v. 0.39; [26]) was applied (mean Phred score ≤ 30; Phred score at 5’ and 3’ ends ≤ 20). All of the obtained reads were trimmed to an equal length of 90 bp and after that mapped to the porcine reference genome with ENSEMBL annotation (Sus_scrofa.11.1.100) using STAR mapper (v. 2.7.1a; [27]) software. StringTie (v. 2.1.1; [28]) pipeline was applied for the annotation and estimation of the expression of porcine genes and uncovered regulatory transcriptionally active regions (TARs). The differentially expressed genes (DEGs) were identified using the DESeq2 tool (v. 1.34.0; [29]) among genes reaching p-adjusted < 0.05 and absolute normalized (log2) fold change (log_2_FC) ≤ 0.54. During the analyses, the batch effect of applying biological material from the same animals in both the adipokine-treated and the control group was adjusted using the limma tool (v. 3.58.1; [30]). The functional analyses of the obtained DEGs (gene ontology and pathway enrichment) were conducted using the KO-Based Annotation System (KOBAS; v. 3.0) tool related to the Kyoto Encyclopedia of Genes and Genomes (KEGG), Gene Ontology (GO), and The Reactome Knowledgebases [31–36] databases. The obtained DEGs were visualized using MA, the Volcano, and heatmap plots with *gplots* Bioconductor package within the R environment (v. 4.1.0; [37]). Raw reads were deposited in the Functional Genomics Data Collection (ArrayExpress) database under the common project accession number E-MTAB-13,773.

### Alternative splicing (AS) analysis

Differentially expressed AS events (DASs) were predicted by the replicate multivariate analysis of transcript splicing software (rMATS; v.2) [38]. Processed reads with 90 bp length were used to estimate the percent of splicing inclusion (PSI). The changes in PSI (∆PSI) between the control groups and apelin-13-treated samples were statistically evaluated by the likelihood-ratio test. DASs were statistically confirmed by false discovery rate (FDR) < 0.05 and absolute ∆PSI value > 0.1. The revealed AS were classified into one of five types, as follows: alternative 3′ splice site (A3SS), alternative 5′ splice site (A5SS), mutually exclusive exons (MXE), retention intron (RI), and skipping exon (SE). DASs were visualized with the *rmats2sashimiplot* Python tool (v. 2.0.4; https://github.com/Xinglab/rmats2sashimiplot).

### Single nucleotide variants (SNV) analysis

The allele-specific expression variants (ASEs) were conducted based on single nucleotide variants (SNVs) within the transcripts obtained through the RNA-Seq reads mapping to the reference genome sequence. The analysis was conducted with the use of the pipeline built with Picard (v. 2.6.0), and rMATS discovery of differential variants in RNA (rMATS-DVR) (v. 1.0.0) software which uses the gold standard genome analysis toolkit (GATK) (v. 3.6.0) [39–41]. The obtained previously BAM files were recalibrated with the use of Picard and the individual rMATS-DVR modules were used for the identification of the potential occurrence of SNVs in all samples and then for the reveal of the differences in SNVs frequencies between the control and apelin-treated groups. The basis of GATK standard parameters (total depth of 8 base coverage > 10, root mean square mapping quality > 40, quality by depth > 2, mapping quality rank sum > -12.5, rank sum test for the relative positioning of reference versus alternative alleles within reads > -8) was applied for excluding the low-quality and disrupted SNVs. Only SNVs with the alternative allele fraction (AAF) > 0 within at least half of the RNA-Seq samples were qualified for further analyses. Changes in the expression of the revealed SNVs between the experimental groups were considered statistically significant with the absolute change of AAF value > 0.1 and false discovery rate (FDR) < 0.05. SNVs located beyond DEGs were excluded from the analysis. The allelic imbalance ratio of ASEs candidates was confirmed using the chi-square (χ^2^) goodness of fit test (*p* < 0.05). The Ensembl VEP web tool was used to establish the exact location of the identified ASE within genes’ regions, as well as to reveal the effect of single nucleotide mutations on the transcription process of the specific proteins [42].

### Quantitative reverse transcription PCR (qPCR) of DEGs (validation method)

To validate the RNA-Seq method, qPCR analysis was conducted. One microgram of RNA was reverse transcribed into cDNA in a total volume of 20 µl with 0.5 µg oligo (dT)_15_ primer (Roche, Switzerland) using the Omniscript RT Kit (Qiagen, USA). The reaction was conducted at 37 °C for 1 h and terminated by incubation at 93 °C for 5 min. The qPCR analysis was carried out with the use of Aria Mx Real-time PCR System (Agilent Technology, USA) as described by Dobrzyn et al. [22]. qPCR reaction conditions, as well as the primer concentrations and sequences of the chosen DEGs and reference genes are detailed in Table 1. The constitutively expressed genes: cyclophilin A (*PPIA*) and Glyceraldehyde-3-Phosphate Dehydrogenase (*GAPDH*) were used as the internal controls to verify the method. Our preliminary studies revealed that the endometrial *PPIA* and *GAPDH* expression was stable during the analysed gestation period. The qPCR amplification efficiencies of the primers designed in this study were 90–110%. The reaction mixture, in a final volume of 20 µL, consisted of 12.5 µL of Sensitive RT HS-PCR Mix SYBR (A&A Biotechnology, Poland), 0.24 µl ROX (reference dye), 20 ng of cDNA, forward and reverse primers, and Rnase-free water. In the negative, non-template controls (NTC), cDNA was substituted by water, or the reverse transcription step was omitted. All reactions were run in duplicates. The specificity of qPCR reactions was confirmed at the end of the experiment by the analysis of the melting curve. The calculation of validated DEGs relative expression was conducted with the use of the comparative cycle threshold method (ΔΔCT) and normalized using the geometrical means of the reference genes’ Ct values. The normality of qPCR data distributions was confirmed by the Shapiro–Wilk test (*p* > 0.05). The results were statistically checked by the Student’s t-test (*p* < 0.05) using Statistica software (Statsoft Inc, USA).

**Table 1:** Primers and reaction conditions of qPCR and RT-PCR used for the validation of the obtained results

	Primer target	Primer sequence	GenBank accession	Amplicon size (nt)	Primer (nM)		Conditions	Reference
**Differentially Expressed Genes (DEGs)**								
	***ALDH7A1***	F: 5’-CTGCTCCAAAGCGAGGAGAA-3’ R: 5’-CCCCGATCATCCGAGACAAG-3’	NM_213926	180	150	Activation: 95ºC – 10 min 35 cycles of: Denaturation (95ºC – 30 s), Annealing (59ºC – 1 min) and Elongation (72ºC – 30 s) Final extension: 72ºC – 7 min		This study
	***HOXA 10***	F: 5’-AAAGAGCGGCCGGAAGAA-3’ R: 5’-ACGCTGCGGCTGATCTCTAG-3’	AF281156	120	500	Activation: 95ºC – 10 min 35 cycles of: Denaturation (95ºC – 30 s), Annealing (59ºC – 1 min) and Elongation (72ºC – 30 s) Final extension: 72ºC – 7 min		[85]
	***IL-1β***	F: 5’-TGCCAACGTGCAGTCTATGG-3’ R: 5’-TGGGCCAGCCAGCACTAG-3’	NM_001146128	82	100	Activation: 95ºC – 10 min 40 cycles of: Denaturation: (95ºC – 15 s) and Annealing: 60ºC – 1 min		[50]
	***IRF8***	F: 5’-TCCTCCTTCTGAATCCGAACC-3’ R: 5’-GAGCAGGACTTGAGCGGAAA-3’	NM_001252427.2	202	50	Activation: 50ºC – 2 min 95ºC – 10 min 40 cycles of: Denaturation (95ºC – 15 s) and Annealing (60ºC – 1 min)		[86]
	***PDYN***	F: 5’-GAGAGGGAGGGTGGAGATTC-3’ R: 5‘-CAAGACGTCCACCTGGATTC-3’	NM_001004040.1	137	375	Activation: 50ºC – 2 min 95ºC – 10 min 40 cycles of: Denaturation (95ºC – 15 s) and Annealing (54 °C–15 s)		[87]
	***PPIA***	F: 5’-GCACTGGTGGCAAGTCCAT-3’ R: 5’-AGGACC CGTATGCTTCAGGA-3’	AY266299	71	300	Activation: 50ºC – 2 min 95ºC – 10 min 40 cycles of Denaturation (95ºC – 15 s) and Annealing: (60ºC – 1 min)		[88]
	***GAPDH***	F: 5’-CCTTCATTGACCTCCACTACATGGT-3’ R: 5’-CCACAACATACGTAG CACCACGAT C-3’	U48832	183	500	Activation: 61ºC – 20 min 95ºC – 10 min 40 cycles of Denaturation (95ºC – 15 s), Annealing: 62ºC – 1 min, and Elongation (72ºC – 1 min)		[89]
**Differentially Expressed Alternative Splicing Events (DASs)**								
	***ADAM15***	F: 5’-TCTGATGCCAGGTGCCAAG-3’ R: 5’-CGGGATTCCAGCTTCAGGG-3’	NM_001252427.2	Skipping: 124	200	Activation: 95ºC – 3 min 40 cycles of Denaturation (95ºC – 30 s), Annealing (62ºC – 1 min), and Elongation (72ºC – 45 s) Final extension: 72 °C–7 min		This study
				Inclusion: 666				
	***DROSHA***	F: 5’- GCCGCTACAGGTCAGATTACG − 3’ R: 5‘- TCCCAACGAGCTCTCTTCTTC − 3’	M_047767682.1	Skipping: 107	200	Activation: 95ºC – 3 min 40 cycles of Denaturation (95ºC – 30 s), Annealing (60ºC – 1 min), and Elongation: 72ºC – 45 s Final extension: 72 °C–7 min		This study
				Inclusion: 200				
	***MAN2C1***	F: 5’- GCCTGTGTGTGTAATGCAAGAG − 3’ R: 5’- ATCCCAGTACAGGGGGACAT − 3’	XM_001924189.4	Skipping: 92	200	Activation: 95ºC – 3 min 40 cycles of Denaturation (95ºC – 30 s), Annealing (62ºC – 1 min), and Elongation (72ºC – 45 s) Final extension: 72 °C–7 min		This study
				Inclusion: 187				
	***SLC22A18***	F: 5’- TCTCGTCAAGGTCATTTCCG − 3’ R: 5’- CAATCCCACCAGGCTGAAGA − 3’	XM_021082630.1	Skipping: 128 Inclusion: 229	200	Activation: 95ºC – 3 min 40 cycles of: Denaturation (95ºC – 30 s), Annealing (62ºC – 1 min), and Elongation (72ºC – 45 s) Final extension: 72 °C–7 min		This study

*ALDH7A1* - aldehyde dehydrogenase 7 family member A1; *HOXA10* - homeobox A10; *IL-1β* - interleukin 1 beta; *IRF8*- interferon regulatory factor 8; *PDYN* - prodynorphin; *PPIA* - cyclophilin A; *GAPDH* - glyceraldehyde-3-phosphate dehydrogenase; *ADAM15* - ADAM metallopeptidase domain 15; *DROSHA* - Drosha ribonuclease III; *MAN2C1* - mannosidase alpha class 2 C Member 1; *SLC22A18* - solute carrier family 22 member 18; F – forward; R – reverse

### Polymerase chain reaction (PCR; differentially expressed DASs validation)

The characterization of SE events in the genes chosen for the validation of DASs was conducted with the use of Labcycler 48s (Syngen Biotech, Poland) and StartWarm HS-PCR Mix (A&A Biotechnology, Poland). The reaction mixture, in a final volume of 25 µL, contained 12.5 µL of Hot Start PCR Mix, primers (forward and reverse), nuclease-free deionized water, and 30 ng of cDNA. In non-template control, cDNA was substituted by water, or the reverse transcription step was omitted. PCR conditions and the primer sequences of chosen DASs are detailed in Table 1. The obtained PCR products were analysed on 1.5% agarose gels containing Midori Green Advance dye (Nippon Genetics Europe, Germany).

## Results

### Sequencing results

The raw data obtained during RNA-Seq have been submitted to the Functional Genomics Data Collection (ArrayExpress) database (https://www.ebi.ac.uk/biostudies/arrayexpress; data accession number: E-MTAB-13,773). Created RNA-seq libraries were sequenced in four biological replicates for both, control and apelin-13 treated groups. HiSeq2500 platform provided 467,751,316 raw paired-end reads, with an average number of 58,47 million reads per sample (Supplementary Table 1). Pre-processing and the removal of adapter sequences delivered 418,477,727 clean reads, from which 402,438,503 reads were uniquely mapped to the reference porcine genome (Sus_scrofa. 11.1.100). The mean percentage distribution of reads aligned to the porcine genome was as follows: 48.42% to coding sequences regions, 24.76% to untranslated regions, 7.95% to introns, and 18.87% to intergenic locations (Supplementary Table 1). In the porcine endometrium, we have identified 23,583 TARs that were expressed in at least half of the examined samples, from which 90 differentially expressed TARs were detected (*p*-adjusted < 0.05) (Supplementary Table 2).

### DEGs and functional annotations (GO, KEGG and Reactome)

**Fig. 1:**
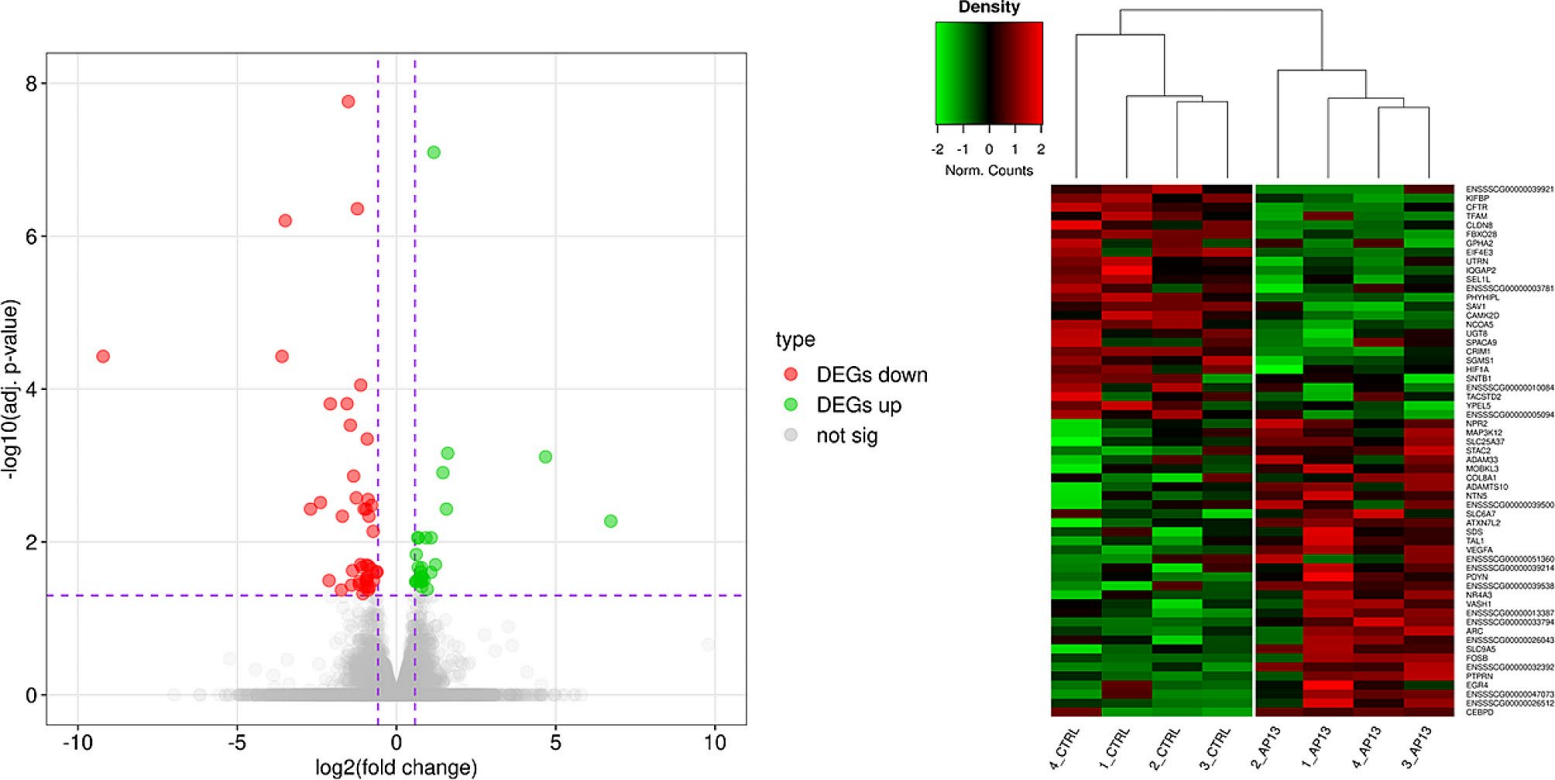
Visualization of the number of genes (**A**), as well as hierarchical clustering heatmap of chosen genes (**B**), whose expression was altered under the influence of apelin-13. The X-axis of volcano plot (**A**) represents logarithmic fold changes in expression (log_2_FC), whereas the Y-axis shows the negative decimal logarithm of the p-values. The horizontal line refers to the negative logarithmic p-value cut-off (*p* = 1.3). The vertical lines mark the fold change cut-offs (log_2_FC > |0.54|). Each column of heatmap (**B**) represents biological replicates of control (CTRL) or apelin-13-treated probes (AP13). Different colours of brackets represent the normalized (Z-score; red-green scale) expression values for DEGs in each biological replicate

**Fig. 2:**
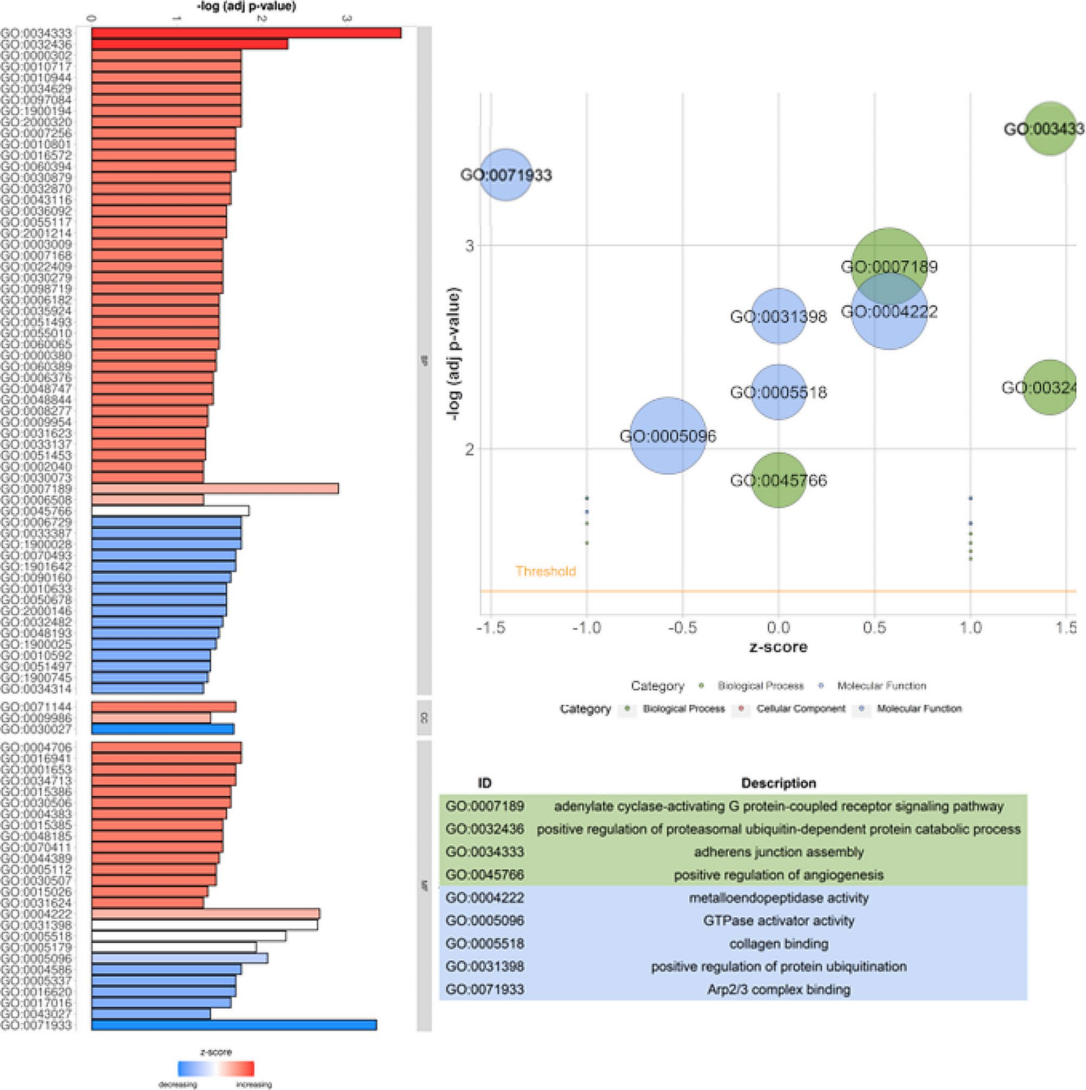
Summary of GO terms enrichment analysis with differentially expressed genes (DEGs). Left panel: The bar plot represents the ratio between up- and down-regulated DEGs. Red bars represent GO terms with the advantage of up-regulated DEGs, whereas blue bars show terms that consist of down-regulated DEGs, mainly. The GO terms are divided into biological process (BP), cellular component (CC), and molecular function (MF) aspects. The length of the bar indicates the individual terms’ statistical significance levels, obtained during the functional analysis. Right panel: The bubble chart shows the dependence of the normalized ratio of up- and down-regulated DEGs (z-score) on the statistical significance level of GO terms. The size of the bubble is proportional to DEGs abundance identified in the study, classified by the term. The names of the selected GO terms are listed in the table below

In the present study, within 90 TARs, 68 DEGs and 22 noncoding differentially expressed transcripts were identified. Figure 1 presents a heatmap and volcano plot of all DEG profiles. Of all DEGs, 39 were up-regulated and 29 were down-regulated under the influence of apelin-13. The log_2_FC values for DEGs ranged from − 6.781 (*AMY2A*) to 3.916 (*SLC6A5*) (Supplementary Table 2). To discover possible functions of 68 annotated DEGs identified in the tissue explants treated with apelin-13, the genes were classified into one of the following GO categories: ‘biological processes’ (BP), ‘cellular components’ (CC) and ‘molecular function’ (MF). DEGs were assigned to 240 GO terms (*p*-adjusted < 0.05). Among them, 145 terms were assigned to BP, 32 to CC, and 63 to MF category (Supplementary Table 3). The visualization of the most involved statistically significant DEGs and their enrichment in ontology terms has been presented in Fig. 2 and Supplementary Fig. 1.

The most of DEGs from the BP category were connected with the ‘integral component of membrane’ (GO:0016021; 10 DEGs), ‘cytoplasm’ (GO:0005737; 9 DEGs), ‘nucleus’ (GO:0005634; 8 DEGs) and ‘plasma membrane’ (GO:0005886; 6 DEGs) (Fig. 2). In BP category, the most interesting DEGs were enriched to the following subcategories: ‘adherens junction assembly’ (GO:0034333; 2 DEGs), ‘positive regulation of angiogenesis’ (GO:0045766; 2 DEGs), ‘signal transduction’ (GO:0007165; 2 DEGs) and ‘intracellular signal transduction’ (GO:0035556; 2 DEGs) (Fig. 2). The most of genes connected with CC category were annotated to ‘nucleoplasm’ (GO:0005654; 4 DEGs), ‘cell surface’ (GO:0009986; 3 DEGs) and ‘extracellular space’ (GO:0005615; 3 DEGs) (Fig. 2). The most enriched GO terms in MF category were ‘metalloendopeptidase activity’ (GO:0004222; 3 DEGs), ‘GTPase activator activity’ (GO:0005096; 3 DEGs), ‘ATP binding’ (GO:0005524; 3 DEGs) and ‘hormone activity’ (GO:0005179; 2 DEGs).

KEGG enrichment analysis revealed modulation of two signalling pathways, activated by products of DEGs: ‘Vascular smooth muscle contraction’ (KEGG:04270, 4 DEGs) and ‘MAPK signalling pathway’ (KEGG04010, 3 DEGs) (Supplementary Table 3; Supplementary Figs. 2 and 3).

### Alternative splicing analysis results

**Fig. 3:**
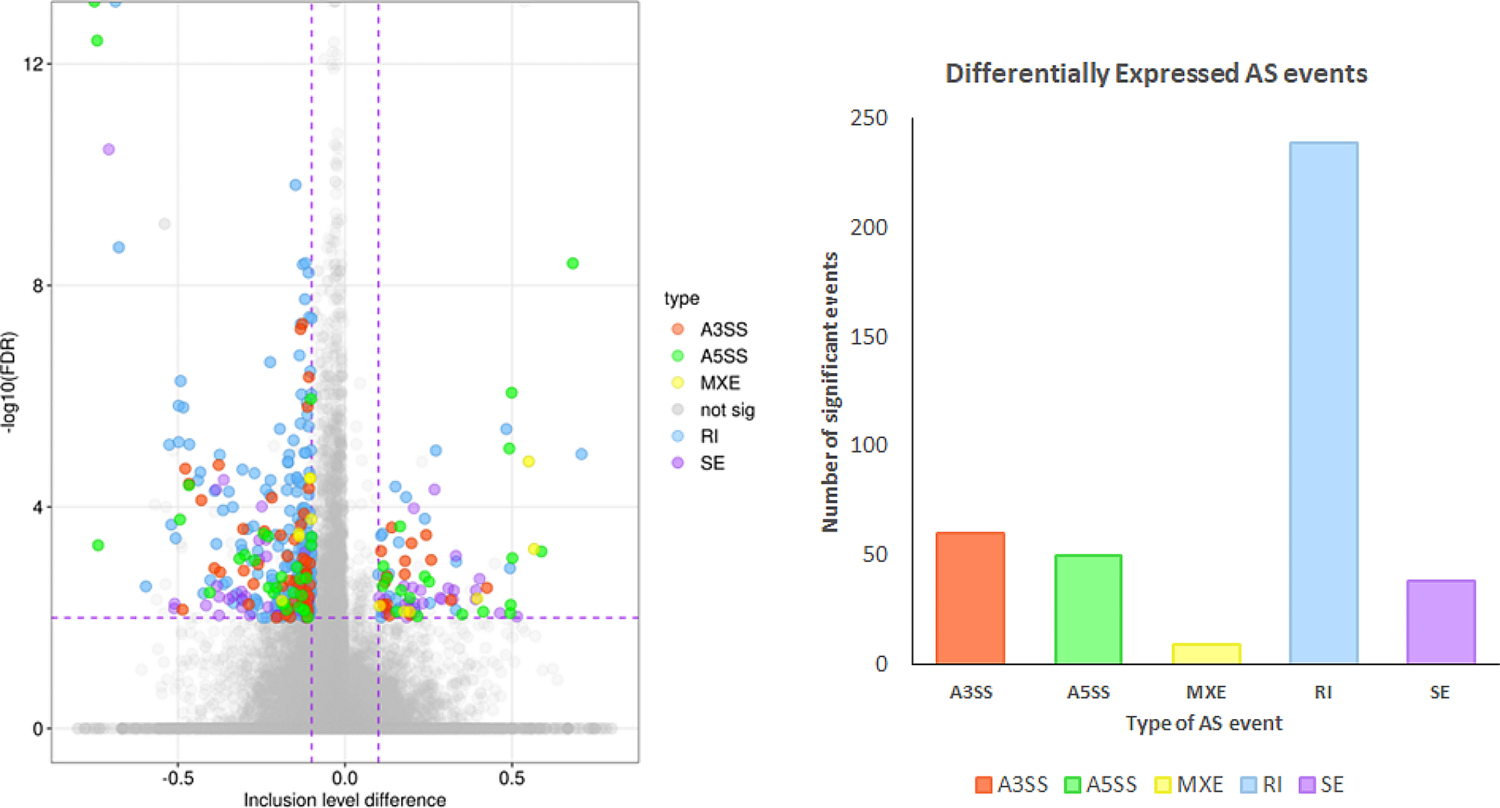
Volcano plot (**A**) presents the inclusion level of differentially alternative splicing events (DASs). Black lines indicate the cut-off thresholds, described in the text. The color dots represent the different types of significant DASs (FDR < 0.05). Inclusion levels of each DASs are presented on the X-axis, and the negative logarithmic adjusted *p* value is presented on the Y-axis. (**B**) Bar plot showing the appearance of DASs. Abbreviations: AS - alternative splicing events, A3SS - alternative 3′ splice site (orange), A5SS - alternative 5′ splice site (green), MXE - mutually exclusive exons (yellow), RI - retention intron (blue), SE - skipping exon (purple), not sig - not significant (grey)

**Fig. 4:**
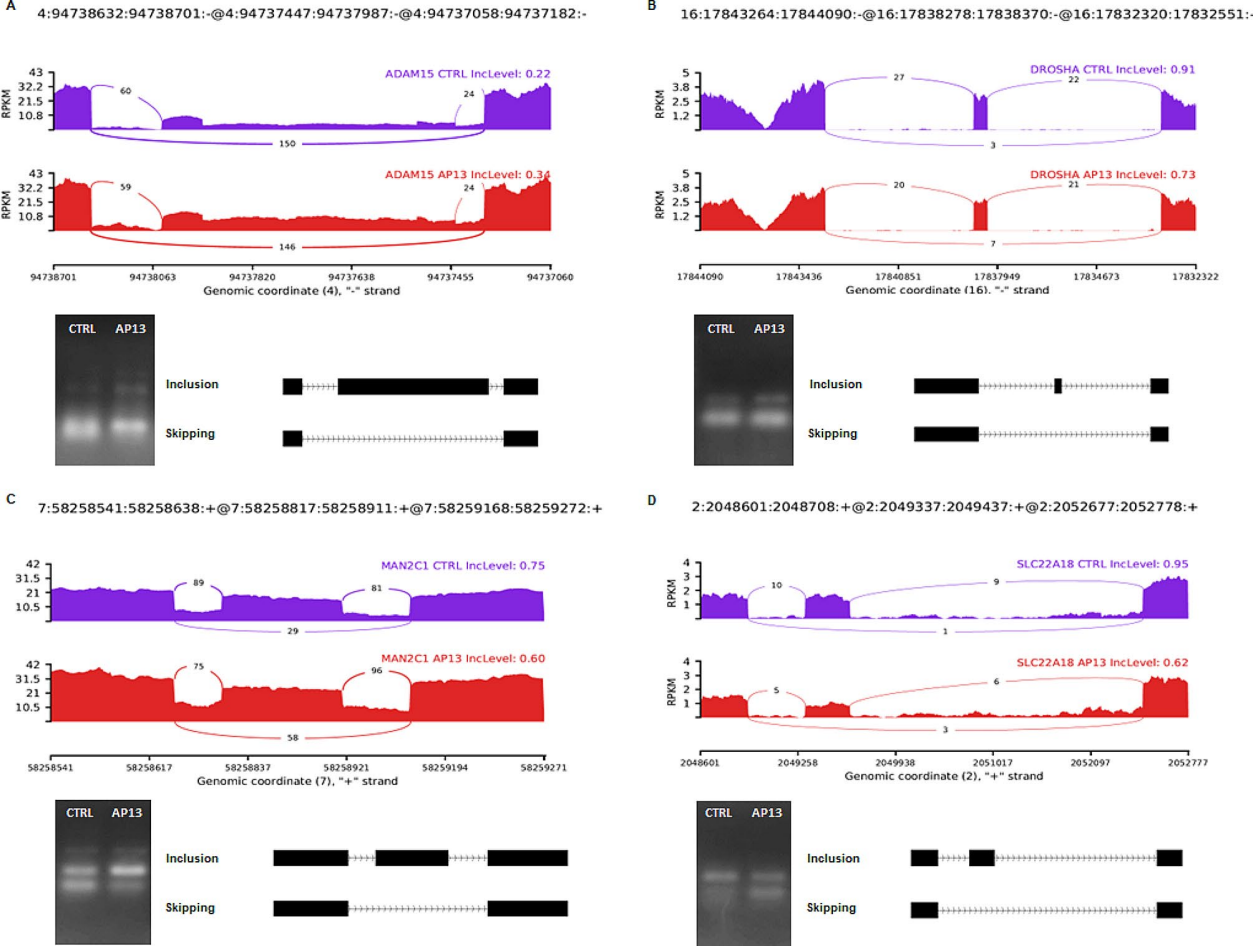
Quantitative visualisation (Sashimi plot) of differential alternative splicing events (DASs) statistically significant in the changes of percentage splicing inclusion (FDR < 0.05 and |ΔPSI|> 0.1) between apelin-13-treated group (AP13; red) and control group (CTRL; purple) samples. The numbers in each upper right corner of the tracks present the percentage of splicing inclusion (PSI) values. Splicing differentiations are covered by the number of reads mapped in the range of junction sites. The upper black tracks show the genomic localisation of splicing events. The plot was generated in ggsashimi Python script. Below of plots, we present the cropped validation results of chosen DASs using the PCR method. The images show the inclusion and skipping exon levels between AP13 and the control group. The full uncropped gel image has been attached as Supplementary Fig. 4. Abbreviations: ADAM15 - ADAM metallopeptidase domain 15, DROSHA - Drosha ribonuclease III, MAN2C1 - mannosidase alpha class 2 C Member 1, SLC22A18 - solute carrier family 22 member 18

The analysis of the obtained results revealed 127,403 AS events, from which 397 were counted to the DASs group revealed after the comparison of control and apelin-13 treated groups. Among all of the revealed events, we identified 61 A3SS, 50 A5SS, 9 MXE, 239 IR, and 38 SE. Figure 3 presents the Volcano plot of the obtained AS events. Figure 4 presents the selected events of AS that occur in ADAM metallopeptidase domain 15 (*ADAM15*), Drosha ribonuclease III (*DROSHA*), mannosidase alpha class 2 C Member 1 (*MAN2C1*), and solute carrier family 22 member 18 (*SLC22A18*) genes. Results obtained during DASs analysis were detailed in Supplementary Table 4.

### Single nucleotide variants analysis results

**Fig. 5:**
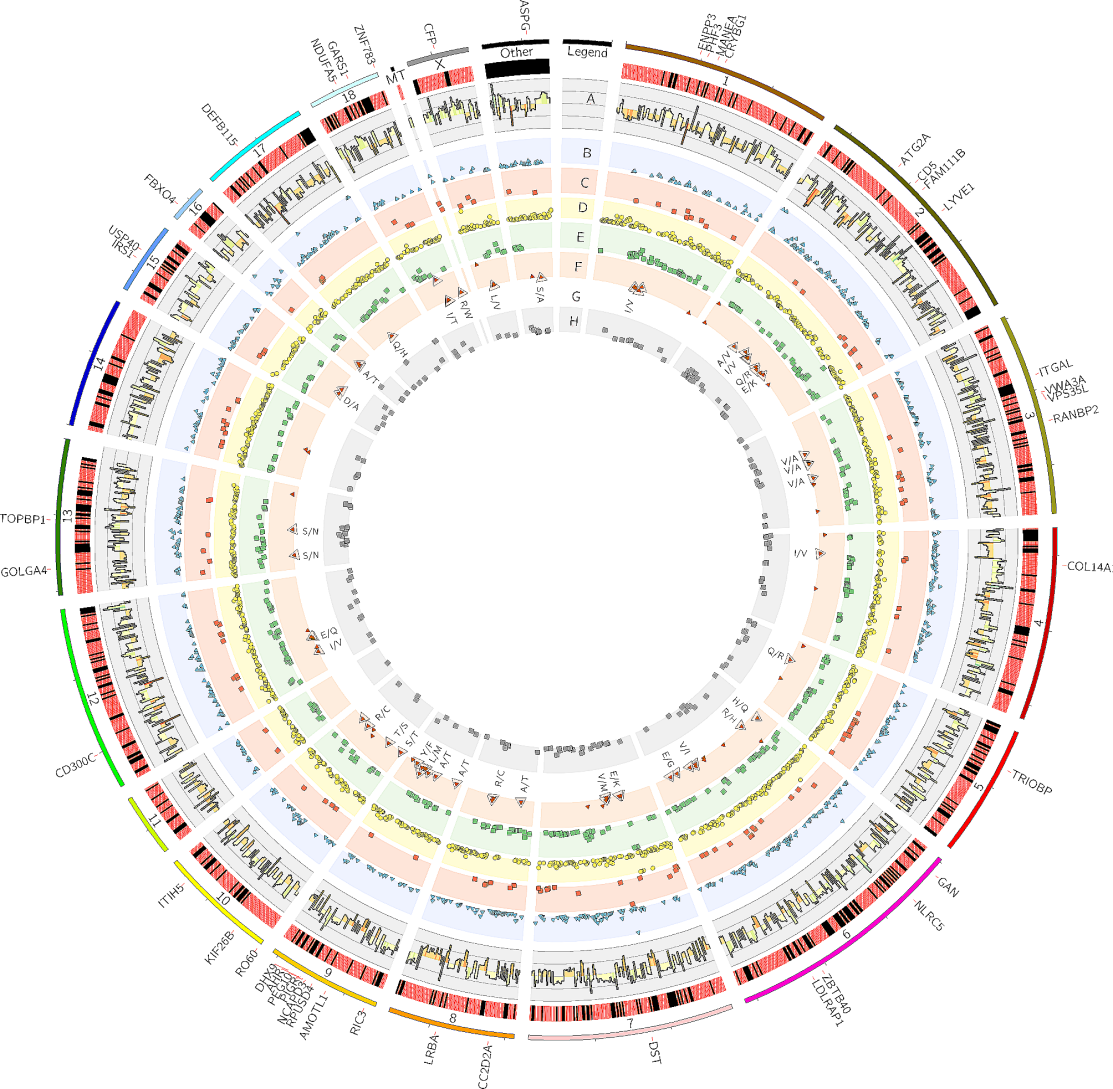
A circular chart presenting the associations between single nucleotide variants in apelin effects on the porcine endometrium on days 15 to 16 of gestation. The outer track represents the gene symbols with alternative allele frequency (AAF) causing missense substitution in CDS. The heatmap presents canonical (A > G and C > T; black) and noncanonical (other; red) substitutions. **A**) The histogram tracks present the changes of AAF (-1 to 1) between apelin effects. The next five scatter tracks present SNV located within the upstream, downstream, and intergenic regions (**B**), causing synonymous substitution (**C**), intron region (**D**), 3 prime and 5 prime UTR region (**E**), causing missense effect (**F**) and within splicing regions (**H**). The G track shows amino acid changes caused by missense substitutions

After filtering out results from eight RNA Seq libraries that did not meet the criteria of GATK standard parameters and ‘AAF > 0’, the obtained SNVs were admitted to further analyses. Among them, 1,818 meet the criteria of the statistically significant allelic imbalance at the same loci (|ΔAAF| > 0.1 and FDR < 0.05) between apelin-13 treated and control samples. Following VEP annotations, 495 ASEs were found to be novel and 1,323 were previously known. Detected ASEs overlapped 4,245 transcripts encoded by 1,695 genes. The summary and consequences of revealed ASEs are summarized in Fig. 5. Among the results revealed by VEP, 564 ASEs were connected with the 3’UTR variant, 83 with the 5’ UTR variant and 942 ASEs belonged to the ‘Downstream gene variant’, a group of variants connected with the sequence variants located at 3’ of a gene. The next group of 531 ASEs belonged to the ‘Upstream gene variant’ group connected with the sequence variants located at 5’ of a gene. Another group of 39 ASEs was identified in the ‘Intergenic region’ and the next groups of 3,286 and 67 ASEs were identified as ‘Intron variant’ and ‘Noncoding transcript exon variant’, respectively. The next groups of 220 and 383 ASEs were identified as ‘Missense variants’ and ‘Splice acceptor variants’. The last two groups of 327 and 3 DASs belonged to the ‘Synonymous variant’ and ‘Stop gained’ groups, respectively. The detailed features of discovered ASEs, their biological impact on genes, and the protein translation process were summarized in Supplementary Table 5.

### Quantitative real-time PCR and PCR analyses results

**Fig. 6:**
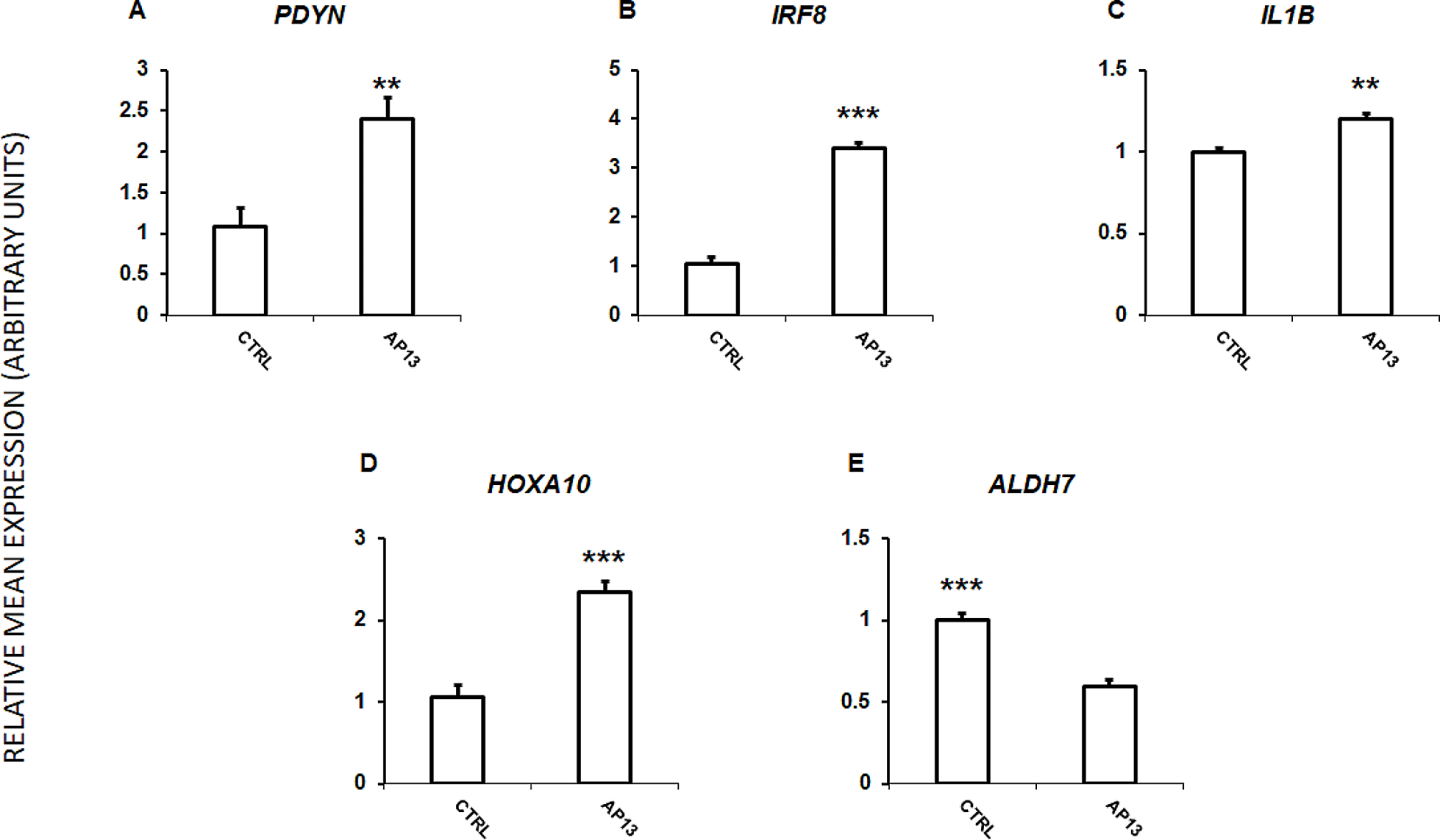
Quantitative real-time PCR validation of RNA-seq results for DEGs in the apelin-13-treated endometrial tissue explants. Validation was performed for (**A**) prodynorphin (PDYN), (**B**) interferon regulatory factor 8 (IRF8), (**C**) interleukin 1 beta (IL1B), (**D**) homeobox A10 (HOXA10), and (**E**) aldehyde dehydrogenase 3 family member B1 (ALDH7; validated genes), as well as for glyceraldehyde-3-phosphate dehydrogenase (GAPDH) and cyclophilin A (PPIA) (reference genes; * *p*-value < 0.05; ** *p*-value < 0.01., *** *p*-value < 0.001)

To validate the obtained RNA-Seq results, five DEGs: prodynorphin (*PDYN*), interferon regulatory factor 8 (*IRF8*), interleukin 1 beta (*IL1B*), homeobox A10 (*HOXA10*), and aldehyde dehydrogenase 3 family member B1 (*ALDH7*) were selected for qPCR experiment. For the validation of AS analysis four DASs were chosen (*ADAM15, DROSHA, MAN2C1*, and *SLC22A18*). The results obtained during qPCR analyses of DEGs, as well as the results of PCR analysis of DASs confirmed data obtained from the RNA-Seq method (Figs. 5 and 6). Validation results confirmed the veracity and accuracy of the RNA-Seq method, as well as the data analysis methods used in the present study.

## Discussion

The present study is the first, to our knowledge, to report the significant influence of apelin-13 on the endometrial global gene expression during the peri-implantation period. In the present study, we revealed that apelin-13 changed the expression of 68 genes. What is surprising, none of the 9,601 identified long non-coding RNA sequences presented a different expression profile after apelin-13 treatment (data not described). Among 68 revealed DEGs, 39 were up-regulated and the expression of 29 was suppressed in the presence of apelin-13. Furthermore, the hormone also affected 397 DASs, as well as 1,818 ASEs.

Analysis of the presented herein data revealed the regulatory influence of apelin-13 on the genes whose products are connected with the regulation of the reproductive functions in the uterus. The analysis of DEGs revealed that the adipokine enhanced the expression of homeobox A10 (*HOXA10*) and interleukin 1β (*IL1B*) genes. The first of the mentioned genes is a member of the transcription factors HOX family, well known for its pivotal role in the regulation of morphogenesis and embryonal tissue differentiation [43]. Mice with a disruption of the *HOXA10* gene are infertile through implantation failure [44]. The drop of *HOXA10* gene expression was also observed in women with adenomyosis and a decreased rate of proper implantation [45] The presence of this transcription factor was also confirmed in pigs, in both, the endometrium and conceptuses, and its expression raised during the peri-implantation period [46]. The product of the next of the revealed genes, proinflammatory IL1β is known for its important role in the immune system functioning and early pregnancy events regulation. IL1β has been observed to be a necessary factor in the process of embryo implantation. In mice, the presence of IL1β receptor antagonist prevented blastocyst adhesion, which was caused by the disruption of the expression of genes encoding integrins that are necessary for the embryo adhesion process [47, 48]. In pigs, during the attachment phase of implantation, conceptuses provoke the acute-phase inflammatory response. IL1β has been suggested to be an important part of the mechanism regulating the maternal immune response to the embryo antigens and, consequently, the pregnancy establishment [49, 50]. The proven stimulatory effect of apelin on the expression of genes whose products are responsible for a proper course of implantation may suggest the regulatory role of this process.

One of the most important aims of the tissue remodelling process during the peri-implantation period is the preparation of the endometrium for embryo attachment. A pivotal part of this process is the development of new vessels which would be able to support the developing embryos with nutrients and a number of signalling molecules and growth factors which ensure proper development of the foetus. The analysis of the obtained results revealed the involvement of apelin in the regulation of the expression of genes that participate in the angiogenesis process. The analysis of gene ontology showed that the apelin enhanced the expression of receptor activity-modifying protein 2 (*RAMP2*) and ADAM metallopeptidase domain 12 (*ADAM12*) genes connected with the *positive regulation of angiogenesis* (GO:0045766). Receptor activity-modifying protein (RAMP) is a group of accessory proteins accompanying GPCR calcitonin receptor-like receptor (CALCRL) which mediates adrenomedullin (AM) actions. AM is an important regulator of vascular development. It was shown that the lack of AM led to mice embryonic or foetal death/loss in the uterus due to the disruption of the vasculature development. The same effect was observed in mice with the deletion of the *RAMP2* gene which suggests its key role in AM actions [51, 52]. What is more, it was indicated that in pigs, the endometrial mRNA content of *RAMP2* was higher during the early gestation period than during the corresponding days of the cycle. Moreover, a significant rise of gene expression in the porcine embryos was reported between days 10 and 16 of gestation. The above results imply an important contribution of *RAMP2* in both, the uterine and embryo angiogenesis processes [53]. What is interesting, the analysis of the obtained DEGs revealed that apelin also enhanced the expression of another gene, calcitonin gene-related peptide (*CALCB*) that belongs to the same family as AM and regulates uterine angiogenesis and foetal growth. CALCB was found to be the next factor responsible for the regulation of foetoplacental growth through its effect on pregnancy-induced vascular remodelling. The disruption of its expression results in foetoplacental growth restriction and probably preeclampsia [54, 55]. The product of the second-mentioned gene connected with *positive regulation of angiogenesis*, *ADAM12* has been also indicated as a crucial factor in the proper uterine angiogenesis process. This member of the disintegrin/metalloprotease family was found to be involved, i.a. in the regulation of muscle cell fusion, as well as in the activation of cell surface integrins [56, 57]. The significant role of ADAM12 in the promotion of the angiogenesis process was indicated in the mice model and the tumour cell lines [58, 59]. Besides these two genes, we also revealed other genes that participate in the process of vasculature development. Apelin treatment enhanced the expression of another member of the ADAM gene family, *ADAM33*, as well as FosB proto-oncogene, AP-1 transcription factor subunit (*FOSB*), and SMAD family member 7 (*SMAD7*) gene expression. The products of *FOSB* and *SMAD7* genes were also found to be the key factors in vessel development [60, 61]. Based on the previously mentioned results and due to the proven participation of apelin in the supporting of angiogenesis by the stimulation of the endothelial cell proliferation in several studies [62–64] we assumed that apelin may play an important role in the promotion of endometrial angiogenesis during the peri-implantation period. The above may also be supported by the explored influence of adipokine on AS events in genes with a proven role in the regulation of angiogenesis, such as Notch receptor 1 (*NOTCH1*) and roundabout guidance receptor 4 (*ROBO4*) [65, 66]. Both of the revealed DASs have been classified as IR. In the case of *ROBO4*, 132 nucleotide IR (ΔPSI = -0.102) between exons 15 and 16 resulted in the appearance of a STOP codon and, as a result, the generation of an inactive isoform of the protein. The ROBO4 protein has been identified as the inhibitor of angiogenesis [65]. Therefore, we suggest that apelin may play as the endometrial angiogenesis promotor through the stimulation of the synthesis of the inactive form of this protein. In opposition to *ROBO4, NOTCH1* has been identified as an essential factor in angiogenesis and vascularisation processes [66]. Herein, we identified 717 nucleotide IR (ΔPSI = -0.139) between exons 27 and 28 of the *NOTCH1* gene which also resulted in the generation of a STOP codon and the loss of protein functionality. One may not exclude, that through the regulation of the functional protein synthesis and, in consequence, reduction of the NOTCH1 signalling, the adipokine may regulate the intensity of the angiogenesis avoiding pathological blood vessel overgrowth.

The process of tissue remodelling, besides the vasculature development, includes also other structural changes which require a proper balance between apoptosis and cell proliferation. Apoptosis is an essential, permanent, dynamic, and interactive biological process when the unnecessary cells are eliminated without triggering inflammatory events [67]. It has been shown that the apelin system may play an inhibitory role in apoptosis regulation, mainly through the inhibition of the PI3K/Akt signalling pathway in the different types of tissues [68–70]. However, some authors also reported the pro-apoptotic action of apelin in pulmonary arterial smooth muscle cells and enterocyte model, Caco-2 cell line [71, 72]. Herein, we indicated the stimulatory effect of the adipokine on the endometrial expression of *TGM3* (Transglutaminase 3) and the inhibitory one on the X-linked inhibitor of apoptosis (*XIAP*) gene. TGM3 is mainly expressed in the small intestine, skin, mucosa, and brain [73]. It has been demonstrated that, through cross-linking of structural proteins, TGM3 is essential for epidermal cells’ terminal differentiation and formation of the cornified cell envelope [74, 75]. The product of the *TGM3* gene has been also identified as an important inhibitor of cell proliferation and enhancer of apoptosis considered a significant anti-tumour factor [76, 77]. The second of the mentioned genes, *XIAP*, is well known for its anti-apoptotic properties, as a direct inhibitor of proteins from the caspases family [78] and its down-regulation is important in the process of endometrial decidualization in rats [79, 80].

The thesis on the regulatory role of apelin in the tissue remodelling process may also be confirmed by the results of DASs analysis. The analysis revealed that apelin altered AS frequency in genes connected with tissue remodelling, such as *ADAM15* and integrin subunit alpha 2b (*ITGA2B*). The product of the *ADAM15* gene is the disintegrin/metalloprotease with the ability to cleave type IV collagen and gelatin [81]. Due to these abilities, ADAM15 has been proposed to be an important factor in the process of extracellular matrix (ECM) reconstruction during the implantation process in mice [82]. The analysis of the obtained DASs indicated SE in the *ADAM15* gene sequence (ΔPSI = -0.118) which results in the promotion of the longer, 858 aa ADAM15 protein isoform. This could enhance the role of ADAM15 in the regulation of cell-cell junctions [83]. The second of the mentioned genes, *ITGA2B*, encodes integrin which was connected with ECM remodelling through the stimulation of type I collagen synthesis and the induction of collagenase gene expression [84]. In this case, the analysis of AS revealed IR between exons 8 and 9 or 15 and 16 (depending on the transcript variant, ΔPSI = -0.403) which leads to the appearance of a STOP codon across the protein sequence and, as a result, synthesis of the inactive protein. It seems that through the promotion of the transcript variants of proteins responsible for the cleavage of ECM components, as well as through the promotion of inactive variants of other proteins, also responsible for the synthesis of different ECM components, apelin may affect the endometrial ECM composition and the degree of its integrity to prepare the tissue for embryo attachment.

## Conclusions

The present work indicates, for the first time, the significant influence of apelin on the global gene expression in the porcine endometrium during the embryo implantation period. The proposed pro-apoptotic action of the adipokine in the endometrium taken together with its participation in the regulation of ECM composition, as well as the regulation of the angiogenic events presented above, indicates clearly that the adipokine may belong to a group of factors responsible for the uterine tissue remodelling during the peri-implantation period.

### Electronic supplementary material

Below is the link to the electronic supplementary material.

Supplementary Material 1

Supplementary Material 2

Supplementary Material 3

Supplementary Material 4

Supplementary Material 5

Supplementary Material 6

Supplementary Material 7

Supplementary Material 8

Supplementary Material 9

Supplementary Material 10

## Data Availability

The raw data obtained during RNA-Seq have been submitted to the Functional Genomics Data Collection (ArrayExpress) database (https://www.ebi.ac.uk/biostudies/arrayexpress; data accession number: E-MTAB-13773).

## Abbreviations

LE: Luminal epithelium
A3SS: Alternative 3′ splice site
A5SS: Alternative 5′ splice site
ADAM12: ADAM metallopeptidase domain 12
ADAM15: ADAM metallopeptidase domain 15
ADAM33: ADAM metallopeptidase domain 33
ALDH7A1: Aldehyde dehydrogenase 7 family member A1
AM: Adrenomedullin
AMY2A: Amylase Alpha 2 A
APJ: Apelin receptor
ASEs: Allele-specific expression variants
BP: Biological processes
CALCB: Calcitonin gene-related peptide
CALCRL: Calcitonin Receptor Like Receptor
CC: Cellular components
DASs: Differentially expressed AS events
DEGs: Differentially expressed genes
DROSHA: Drosha ribonuclease III
ECM: Extracellular matrix
FDR: False discovery rate
FOSB: FosB Proto-Oncogene, AP-1 Transcription Factor Subunit
FSH: Follicle-stimulating hormone
GAPDH: Glyceraldehyde-3-phosphate dehydrogenase
KEGG: Kyoto Encyclopedia of Genes and Genomes
GO: Gene Ontology
HOXA10: Homeobox A10
IL1β: Interleukin 1 beta
IRF8: Interferon regulatory factor 8
ITGA2B: Integrin subunit alpha 2b
LH: Luteinizing hormone
MAN2C1: Mannosidase alpha class 2 C Member 1
MF: Molecular function
MXE: Mutually exclusive exons
NOTCH1: Notch receptor 1
PDYN: Prodynorphin
PPIA: Cyclophilin A
PSI: Percent of splicing inclusion
qPCR: Quantitative real-time PCR
RAMP2: Receptor activity-modifying protein 2
RI: Retention intron
RIN: RNA integrity number
RNA-seq: RNA next-generation sequencing
ROBO4: Roundabout guidance receptor 4
SE: Skipping exon
SLC22A18: Solute carrier family 22 member 18
SLC6A5: Solute Carrier Family 6 Member 5
SMAD7: SMAD family member 7
SNV: Single nucleotide variants
T: Testosterone
TARs: Transcriptionally active regions
TGM3: Transglutaminase 3
WAT: White adipose tissue
XIAP: X-linked inhibitor of apoptosis
