# Correction: Apelin-13 modulates the endometrial transcriptome of the domestic pig during implantation

**DOI:** 10.1186/s12864-024-10585-8

**Published:** 2024-07-25

**Authors:** Kamil Dobrzyn, Marta Kiezun, Grzegorz Kopij, Barbara Zarzecka, Marlena Gudelska, Katarzyna Kisielewska, Ewa Zaobidna, Karol G. Makowczenko, Cecilia Dall’Aglio, Tadeusz Kamiński, Nina Smolińska

**Affiliations:** 1 Faculty of Biology and Biotechnology, University of Warmia and Mazury in Olsztyn, Oczapowskiego 1A, Olsztyn, 10-719 Poland; 2 School of Medicine, Collegium Medicum, University of Warmia and Mazury in Olsztyn, Aleja Warszawska 30, Olsztyn, 10-082 Poland; 3 Institute of Animal Reproduction and Food Research of Polish Academy of Sciences, Department of Reproductive Immunology and Pathology, Tuwima 10, Olsztyn, 10-748 Poland; 4 Department of Veterinary Medicine, University of Perugia, Via San Costanzo 4, Perugia, 06126 Italy

**Correction:** ***BMC Genomics*** **25, 501 (2024)**

10.1186/s12864-024-10417-9

Following publication of the original article it was reported that the Funding declaration was missing. The missing declaration is the following:

**Funding**.

This research was supported by the National Science Centre (projects no: 2018/31/N/NZ9/00544 and 2019/03/X/NZ9/00213).

The publication was written as a result of the author’s internship in the Department of Veterinary Medicine, University of Perugia, Italy, co-financed by the European Union under the European Social Fund (Operational Program Knowledge Education Development), carried out in the project Development Program at the University of Warmia and Mazury in Olsztyn (POWR.03.05. 00-00-Z310/17).

The original article has been updated to include this declaration.

