# Supplementary figures and images for: Apelin-13 modulates the endometrial transcriptome of the domestic pig during implantation
Source: BMC Genomics. 2024 May 21;25:501. doi: 10.1186/s12864-024-10417-9 (PMC11106924; doi:10.1186/s12864-024-10417-9)

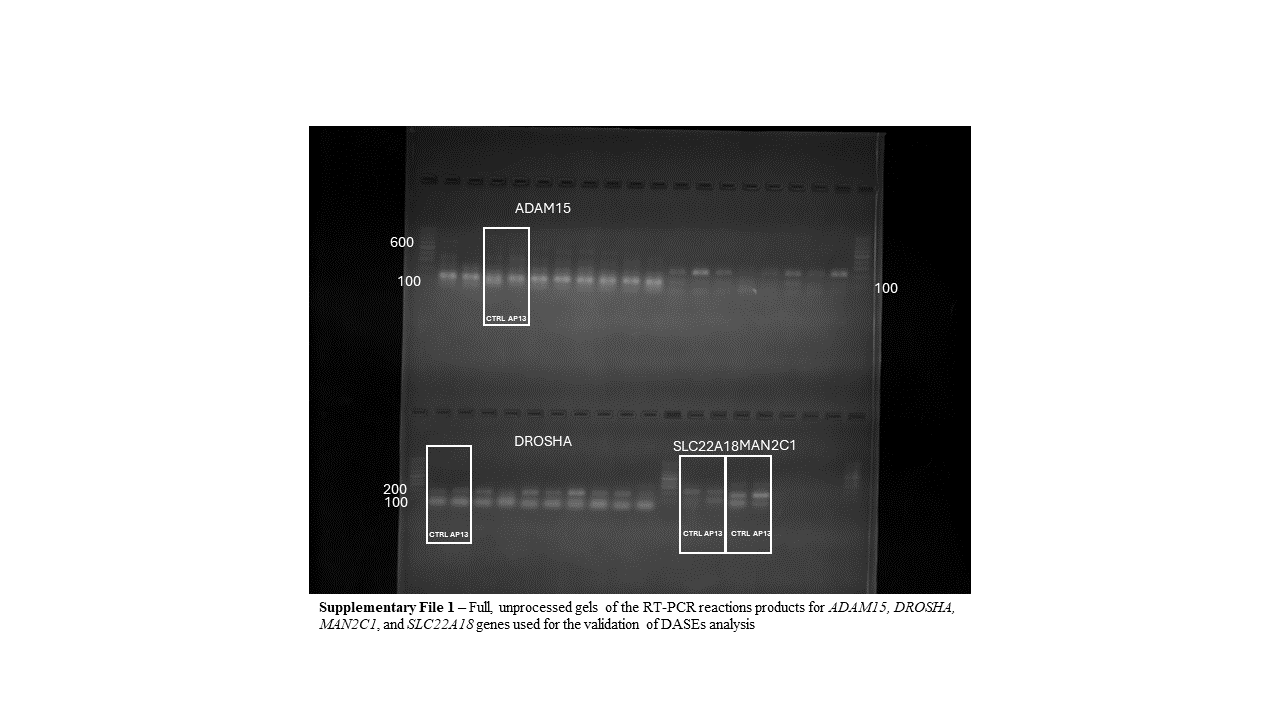

Supplement: Supplementary file 4 — Supplementary Material 4 [file 12864_2024_10417_MOESM4_ESM.png]

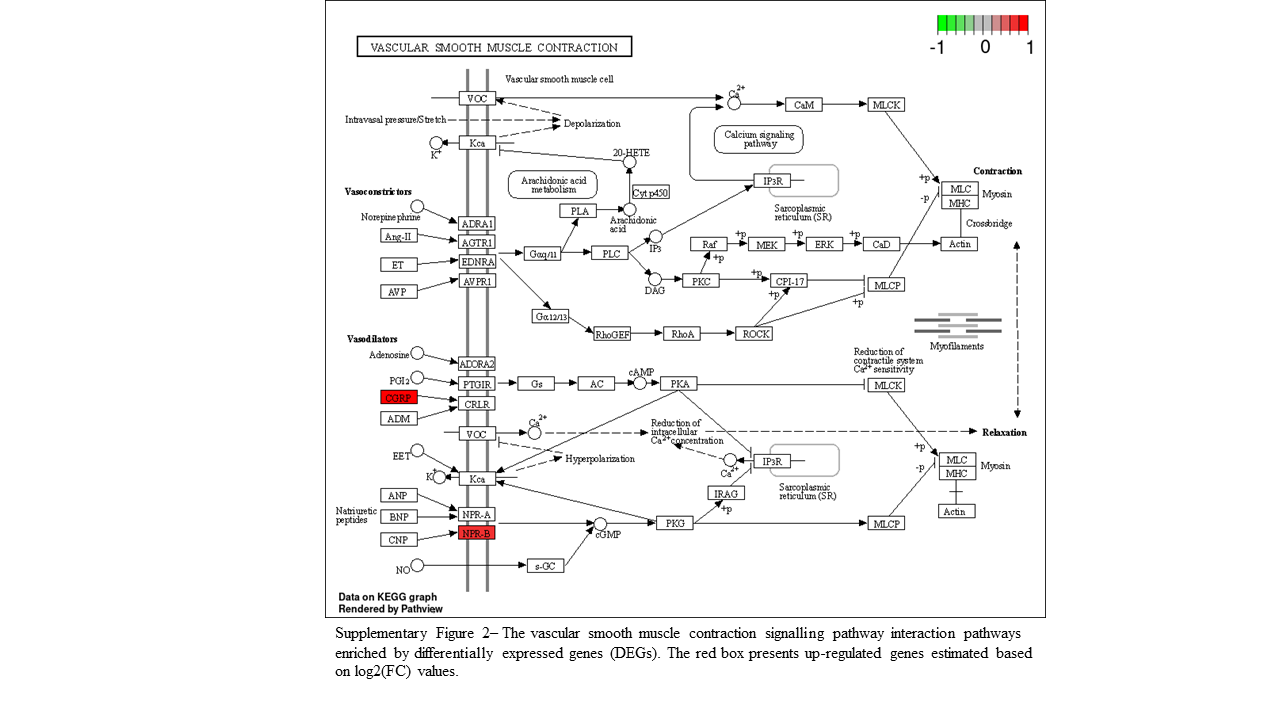

Supplement: Supplementary file 5 — Supplementary Material 5 [file 12864_2024_10417_MOESM5_ESM.png]

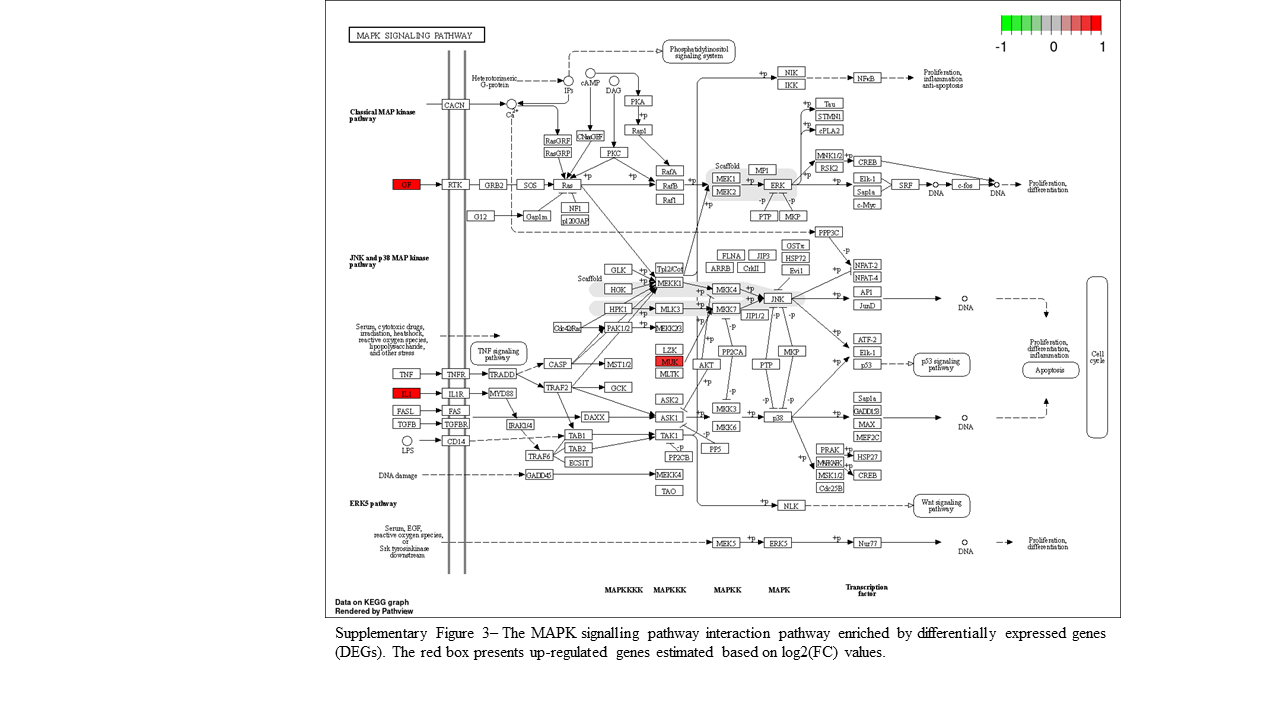

Supplement: Supplementary file 7 — Supplementary Material 7 [file 12864_2024_10417_MOESM7_ESM.png]

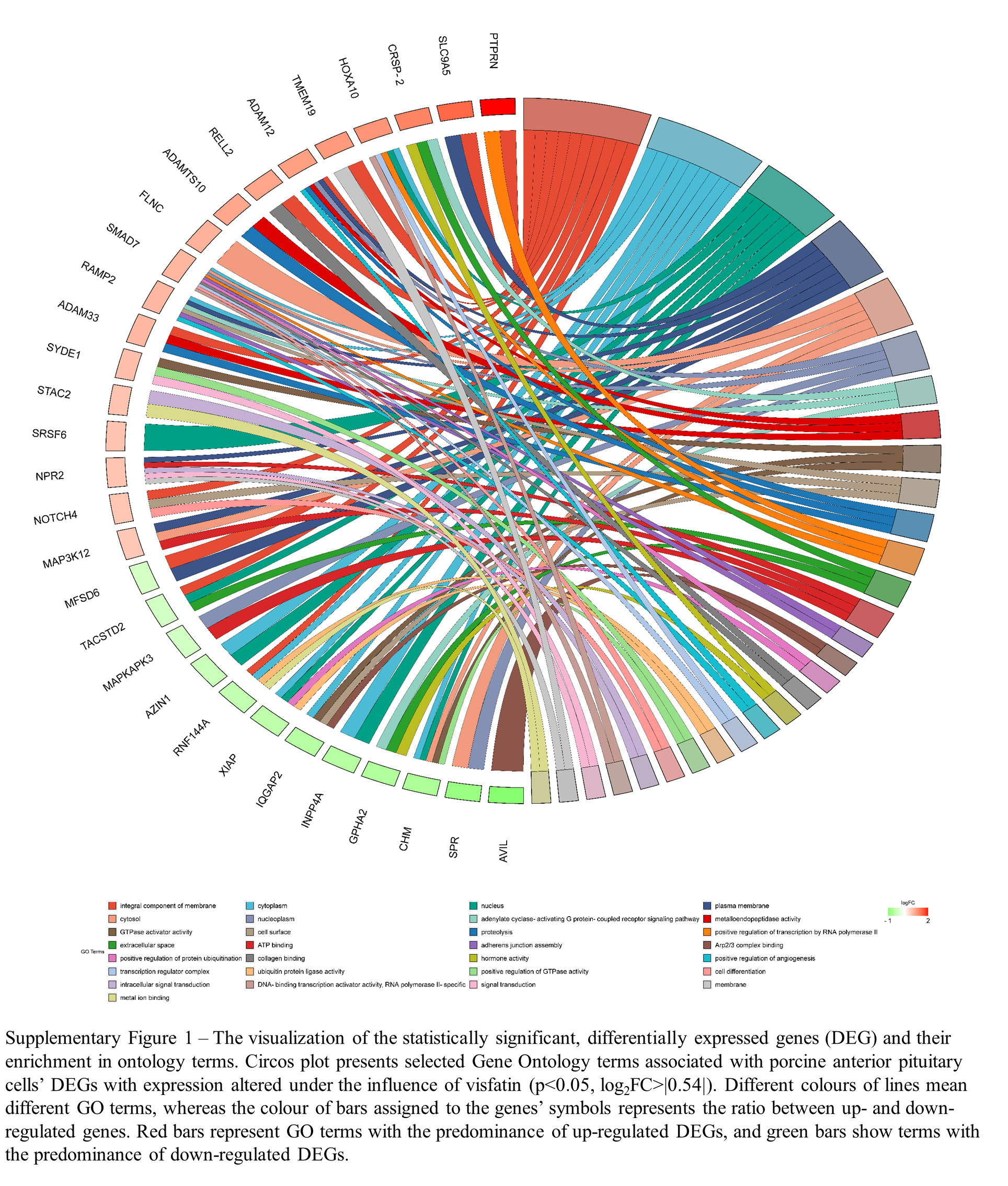

Supplement: Supplementary file 9 — Supplementary Material 9 [file 12864_2024_10417_MOESM9_ESM.png]

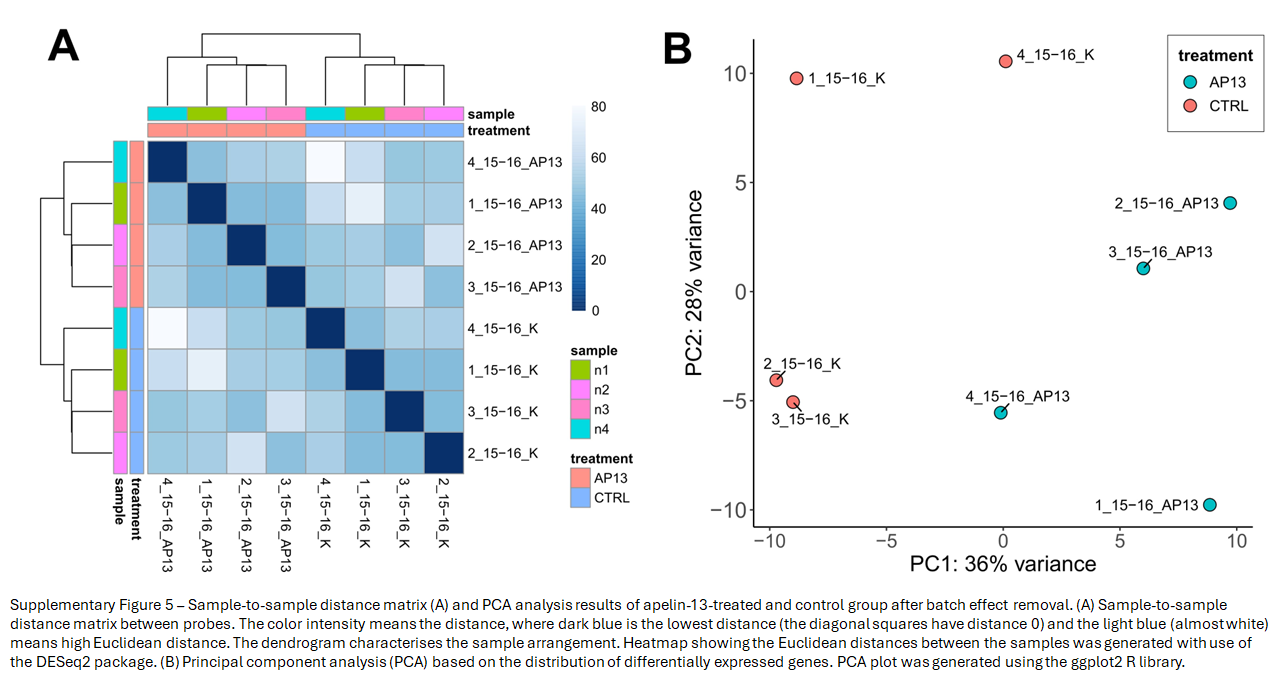

Supplement: Supplementary file 10 — Supplementary Material 10 [file 12864_2024_10417_MOESM10_ESM.png]
